# Forage as a Primary Source of Mycotoxins in Animal Diets

**DOI:** 10.3390/ijerph8010037

**Published:** 2010-12-28

**Authors:** Jiří Skládanka, Jan Nedělník, Vojtěch Adam, Petr Doležal, Hana Moravcová, Vlastimil Dohnal

**Affiliations:** 1 Faculty of Agronomy, Mendel University in Brno, Zemedelska 1, CZ-613 00 Brno, Czech Republic; E-Mails: vojtech.adam@mendelu.cz (V.A.), dolezal@mendelu.cz (P.D.); 2 Agriculture Research, Ltd. Troubsko, Zahradní 1, CZ-664 41 Troubsko, Czech Republic; E-Mails: nedelnik@vupt.cz (J.N.), moravcova@vupt.cz (H.M.); 3 University of Jan Evangelista Purkyně in Ústí nad Labem, Hoření Street 13, CZ-400 96 Ústí nad Labem, Czech Republic; E-Mail: vlastimil.dohnal@ujep.cz

**Keywords:** mycotoxins, deoxynivalenol, zearalenone, fumonisins, aflatoxins, forage, grass, contamination

## Abstract

The issue of moulds and, thus, contamination with mycotoxins is very topical, particularly in connexion with forages from grass stands used at the end of the growing season. Deoxynivalenol (DON), zearalenone (ZEA), fumonisins (FUM) and aflatoxins (AFL) are among the most common mycotoxins. The aim of the paper was to determine concentrations of mycotoxins in selected grasses (*Lolium perenne*, *Festulolium pabulare*, *Festulolium braunii*) and their mixtures with *Festuca rubra* an/or *Poa pratensis* during the growing season as a marker of grass safety, which was assessed according to content of the aforementioned mycotoxins. During the growing season grass forage was contaminated with mycotoxins, most of all by DON and ZEA. The contents of AFL and FUM were zero or below the limit of quantification. Moreover, the level of the occurrence of mould was quantified as ergosterol content, which was higher at the specific date of cut. All results were statistically processed and significant changes were discussed.

## 1. Introduction

Microorganisms in the phyllosphere of grasses are influenced appreciably by changes in grassland management, particularly by the transition from intensive management to extensification due to reduced cutting frequencies and lower fertilizer applications [1–3]. In late autumn, the vegetation of pasture plants gradually decreases and weather conditions stimulate the development of microscopic fungi [4,5], which, in consequence, may lead to the formation of mycotoxins [6]. These metabolites can cause economic losses in animal production if consumed and decrease meat quality [7]. The issue of moulds is very topical, namely in connexion with forages from grass stands used at the end of the growing season. There are considerable differences amongst the species [8,9]. Mould-resistance species include *Festuca arundinacea* and its hybrids [10]. Ergosterol ranks amongst the main sterols produced by lower and higher fungi [11]. Its occurrence in other organisms is very limited, and negligible concentrations of ergosterol in dry matter (DM) were only detected in some bacteria and yeasts. Due to this fact, it is possible in practice to associate the occurrence of this sterol with the presence of moulds in an analyzed sample [12].

Deoxynivalenol (DON), zearalenone (ZEA), fumonisins (FUM) and aflatoxins (AFL) are among the most frequently encountered mycotoxins [13,14]. Trichothecenes, such as 2-T toxin, HT-2 toxin, deoxinivalenol or nivalenol, are very large family of chemically related sesquiterpenic mycotoxins produced by various species of *Fusarium*, *Myrothecium*, *Trichoderma*, *Trichothecium*, *Cephalosporium*, *Verticimonosporium* and *Stachybotrys* [15,16].

Deoxynivalenol (DON) is a natural-occurring mycotoxin mainly produced by *Fusarium graminearum* [17]. It is also know as vomitoxin due to his strong emetic effects after consumption, because it is transported into the brain, where it affects dopaminergic receptors. The emetic effects of this mycotoxin were firstly described in Japanese men consuming mouldy barley containing *Fusarium* fungi [18,19]. DON is probably the best known and most common contaminant of grains and their subsequent products. Its occurrence in food and feed represent more than 90% of the total number of samples and it is a potential marker of the occurrence of other mycotoxins [20].

Zearalenone (ZEA), also known as RAL and F-2 mycotoxin, is a potent estrogenic metabolite produced by some *Fusarium* species, which commonly infect cereal crops. Due to its estrogenic activity, zearalenonee is known to disturb the ovulation cycle and reduce litter size in domestic animals, particularly in swine [21–25]. Apart from the direct impact on ruminants, the contaminated forage affects rumen microorganisms, too [26,27].

Fumonisins (FUM) are produced by some fungi of genus *Fusarium* (especially *F. moniliforme* and *F. proliferatum*). They cause several types of disorders of livestock. Fumonisins can be found in cereals (especially in maize corns and products based on maize) and rice. Distinctive carcinogenic effects have been described, especially formation of oesophagus.

Aflatoxins are toxic secondary metabolites produced by some fungi, especially of genus *Aspergillus* (above all by two species—*Aspergillus flavus* and *Aspergillus parasiticus*). These mycotoxins are toxic for homoeothermic animals including human and induce mycotoxicosis called aflatoxicosis. Aflatoxins called B1, B2, G1 and G2 are the most investigated. There have been identified several ways of poisoning of animals, mainly due to consumption of mouldy wet fodder. When ruminants are fed mouldy fodder, mycotoxins can pass into milk and poison young ones. Presence of aflatoxin is largely associated with commodities produced in the tropics and subtropics, such as groundnuts, other edible nuts, figs, spices and maize. Aflatoxins may be acutely toxic, carcinogenic, mutagenic and teratogenic. These compounds are primarily metabolised in the livers of vertebrates [28,29].

New technologies and procedures which would be acceptable for the easy, rapid and safe removal of mycotoxins from environment, are still being researched. Technologies for aflatoxin isolation with bentonite, modified bentonite as well as chitosan have been tested [30–35].

The aim of the paper was to assess the safety of selected grasses (*Lolium perenne*, *Festulolium pabulare*, *Festulolium braunii*) and their mixtures with *Festuca rubra* and/or *Poa pratensis* during the growing season. Safety of the grass was assessed according to content of the following mycotoxins: zearalenone, aflatoxin, deoxynivalenol, and fumonisin. Level of the occurrence of mould was quantified as ergosterol content.

## 2. Experimental Section

### 2.1. Experimental Localation

**Figure 1:**
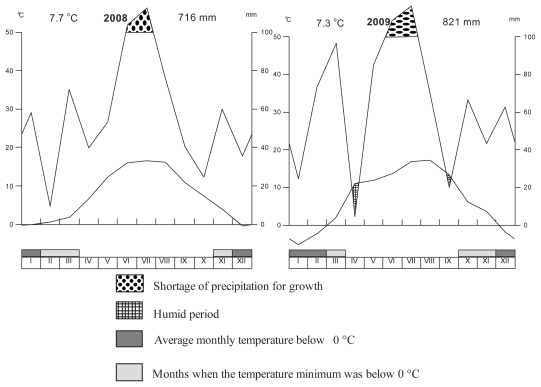
Temperature and precipitation at the Research Station Vatin in 2008 and 2009.

The small-plot experiment was conducted in the Research Station of Fodder Crops in Vatín, Czech Republic (49°31′N, 15°58′E) and established in 2007 at the altitude of 560 m a.s.l. In 1970–2000, mean annual precipitation was 617 mm and mean annual temperature was 6.9 °C. Temperature and precipitation at the Research Station Vatin in 2008 and 2009 are shown in Figure 1. Soil type used in our experiments was Cambisol as a sandy-loam on the diluvium of biotic orthogneiss. Soil nutrient content was in year of observation 89.1 mg kg^−1^ P, 231.6 mg kg^−1^ K, 855 mg kg^−1^ Ca and pH was 4.76.

### 2.2. Experimental Design

A split plot design with 1.5 × 10 m plots was used. The main plots were species and the subplots were harvest dates. The experiments were carried out in triplicate. The first evaluated factor was species: *Lolium perenne* (cv. Kenatur), *Festulolium pabulare* (cv. Felina), *Festulolium braunii* (cv. Perseus), mixtures of these species with *Festuca rubra* (cv. Gondolin) and/or P*oa pratensis* (cv. Slezanka). The share of *Festuca rubra* and/or *Poa pratensis* in the mixture was 15%. The second evaluated factor was harvest date. In summer, the grass stand was cut in June and also in July. Subsequently autumn harvest dates were October and/or November and/or December. The observation took place in two year: 2008 and 2009.

Pure stands of each species were sown with 30 kg ha^−1^ seeds and each mixture was sown at 37.5 kg ha^−1^. The experimental plots were fertilized with 50 kg ha^−1^ N. The plots were harvested by self-propelled mowing machine with an engagement of 1.25 m. Harvested area was 12.5 m^2^.

### 2.3. Detected Parameters

An ELISA method was applied for estimation of the contents of the mycotoxins. The ELISA assay test is a competitive direct enzyme-linked immunosorbent assay for the quantitative analysis of DON, ZEA, FUM and AFL in different commodities as grains, feed *etc.* The test kits are provided in a microwell format which allows the user to obtain exact concentrations in parts-per-billion (ppb) of toxins. Free toxin molecules in the samples and control are allowed to compete with enzyme-labelled toxins (conjugate) for the antibody binding sites. After a wash steps substrate is added, which reacts with the bound conjugate to produce colour. The test is read in a microwell reader. The optical densities of the control form the standard curve, and the sample optical densities are plotted against the curve to calculate the exact concentration of toxins [36]. The determination of ergosterol was performed on a Zorbax SB-C18 reverse phase chromatographic column (4.6 × 30 mm with a particle size of 1.8 μm; Agilent Technologies, USA). HPLC instrument used was from Agilent Technologies. The separation was carried out at a laboratory temperature using isocratic elution—mobile phase with a composition of methanol/water (97.5/2.5, v/v) at a volumetric velocity of 0.6 mL/min. Ergosterol was detected with UV detector at 282 nm [37,38].

### 2.4. Statistical Analyses

Data were processed using the STATISTICA.CZ Version 8.0 (Czech Republic). Results are expressed as means (x), which are supplement about standard error of mean (s.e.). The obtained results were further analyzed using ANOVA.

## 3. Results and Discussion

### 3.1. Characterization of Species Included in the Study

**Figure 2:**
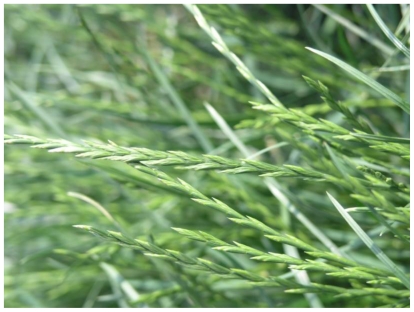
Inflorescence of *Lolium perenne*.

**Figure 3:**
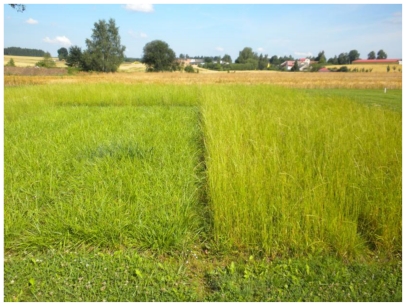
Stands of *Festulolium pabulare* (left) and *Festulolium braunii* (right) before harvesting in late July.

**Figure 4:**
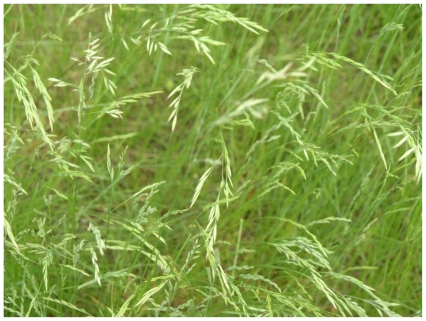
Inflorescence of red fescue (*Festuca rubra*).

**Figure 5:**
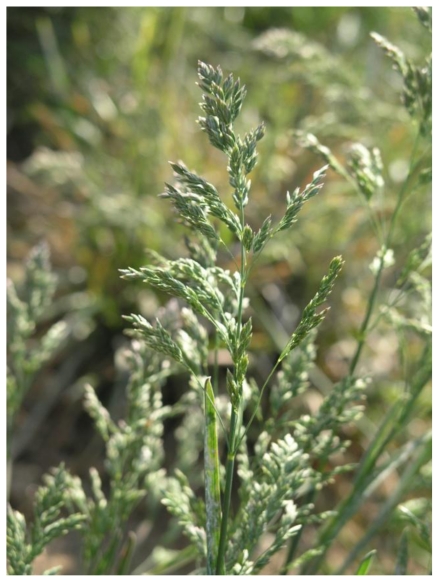
Inflorescence meadow grass leaf infected with mildew.

*Lolium perenne* (Figure 2) is a bunchgrass. The species is demanding of moisture and nutrients and easily undergoes freezing. It is a highly valuable forage grass with a high soluble carbohydrate content. The disadvantage is its susceptibility to fungal diseases. On the contrary *Festulolium pabulare*, resulting from crossbreeding between *Festuca arundinacea* and *Lolium multiflorum*, shows higher resistance to fungal diseases. This species has short rhizomes, is drought resistant and capable of being dotted under low temperatures. Leaves dry easily in autumn and, unlike *Lolium perenne*, are not so susceptible to rotting. *Festulolium braunii* is derived from crossbreeding of *Lolium multiflorum* and *Festuca pratensis*. Its endurance is limited to a period of five years. There is high quality forage, but susceptibility to fungal diseases. Unlike the above mentioned species, this one belongs to the spring grass class (Figure 3), thus, it tends to ear well in the second cut. *Festuca rubra* can be included in the leptomorph grasses group (Figure 4), which display slow development. This species is commonly used to fill the gaps in vegetation by its long rhizomes. *Poa pratensis* is similar to this species (Figure 5), and, unlike *Festuca rubra*, provides higher quality forage.

### 3.2. The Content of Mycotoxins and Statistical Evaluation of the Data

Species did not have a significant effect on the content of mycotoxins (Table 1), but it is clear that *Festulolium pabulare* had lower amounts of ZEA in comparison with other species, however, the difference was not statistically significant.

**Figure 6:**
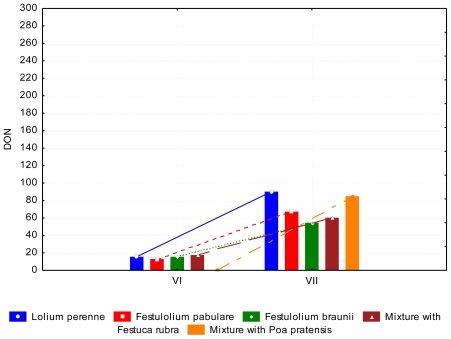
Deoxynivalenol (DON) content (ppb) in summer period, depending on the grass spesies and date of collection.

The resistance of this species to this kind of fungal diseases is shown in the content of ergosterol. Its content was lower in *Festulolium pabulare* (*P* < 0.05) than in *Festulolium braunii*. Ergosterol content gradually increased (*P* < 0.05) from June till December. On the other hand, contents of mycotoxins such as DON in July (*P* < 0.05) and ZEA (P < 0.05) in July and October were significantly higher. Despite the high (*P* < 0.05) content of ergosterol in November and December, low (*P* < 0.05) contents of ZEA were determined in these months. This may be associated with a decrease in temperature when the mould reduces the production of mycotoxins, which are generally a response to stress associated with higher temperatures. The content of DON ranged from 35.07 to 52.78 ppb in the evaluated species in summer (Figure 6).

**Figure 7:**
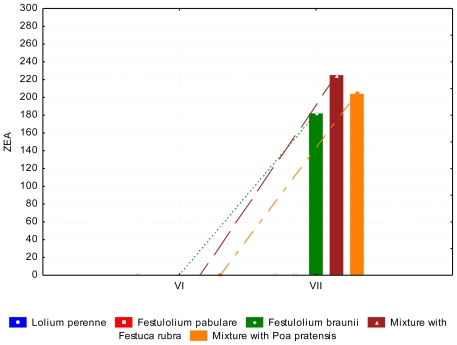
Zearalenone (ZEA) content (ppb) in summer period, depending on the grass species and date of collection.

**Figure 8:**
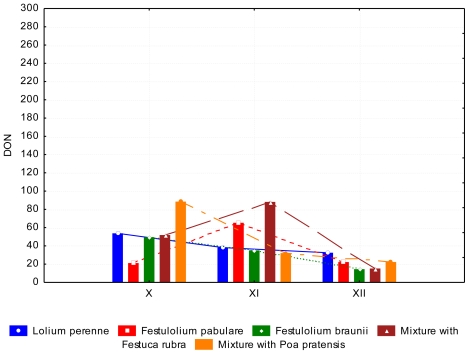
Deoxynivalenol (DON) content (ppb) at the end of growing season, depending on the grass species and date of collection.

**Figure 9:**
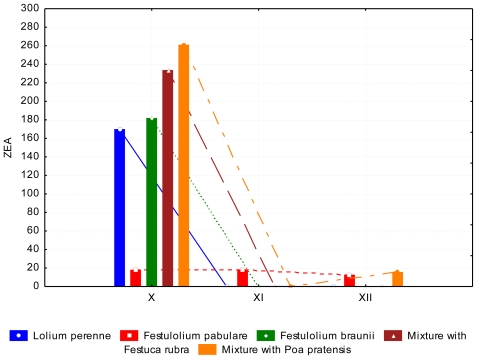
Zearalenone (ZEA) (ppb) at the end of growing season, depending on the grass species and date of collection.

A lower (*P* < 0.05) content of DON occurred in June than in July. The content of ZEA in *Lolium perenne* and *Festulolium pabulare* (Figure 7) was under the limit of quantification (<LOQ). The highest content of ZEA was determined in mixtures with *Festuca rubra* and *Poa pratensis*, respectively. (102.07 ppb and 112.52 ppb, respectively). The major difference between species was not significant because of the high standard errors of the means. The contents of AFL and FUM were zero or under the limit of quantification. This phenomenon was observed in summer and autumn samples. Samples of grasses in autumn contained comparable amounts of DON as summer samples (Figures 8 and 9). During of autumn the content of DON decreased (*P* < 0.05). Differences between years of observation are also significant (*P* < 0.05). The content of ZEA was lowest at *Festulolium braunii*. Content of ZEA decreased from October to December (*P* < 0.01), like for DON. The reason for the low production of mycotoxins can be the decreasing temperatures when mycotoxins are not produced while warm weather during autumn is suitable for mycotoxin production. The effect of not only biotic but also abiotic factors on the production of mycotoxins was observed and discussed by DeNijs *et al*. [39], and Engels and Krämer [40]. The content of ZEA was considerable higher (173.0 ppb) in October than in summer (122 ppb and <LOQ, respectively). According to D’Mello [41], a zearalenone concentration ranging from 0.2–1.0 mg kg^−1^ is even toxic for rodents. Forage with a zearalenone content higher than 0.5 mg kg^−1^ is not advised for feeding [42].

**Figure 10:**
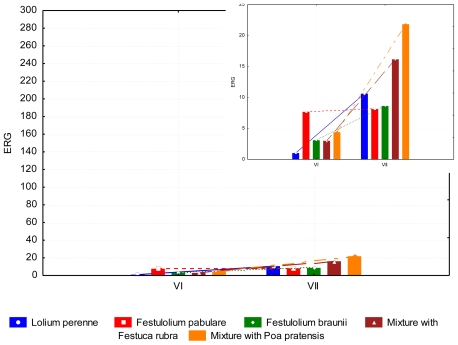
ERG content (mg kg^−1^ dry weight) in summer period, depending on the grass species and date of collection.

Given the fact that fungi are able to create a number of mycotoxins, one mould species can form multiple mycotoxins. In addition mycotoxins are synthesized under stressful conditions, thus, the presence of ZEA and DON does not reflect the current state of content of mould. The content of ergosterol indicates in this context the presence of fungus in all samples. Ergosterol content was lowest in June (3.84 mg kg^−1^ dry weight). During the summer no difference between the evaluated grasses was observed (Figure 10).

**Figure 11:**
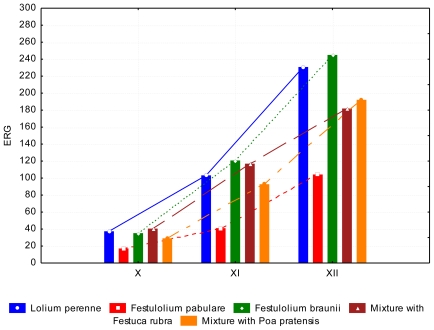
ERG content (mg kg^−1^ dry weight) at the end of growing season, depending on the grass species and date of collection.

Obvious differences between the species were determined in the fall (Figure 11). Even in the case of mycotoxin contamination *Festulolium pabulare* was less contaminated. The low content of ergosterol (*P* < 0.05) statistically confirms *Festulolium pabulare* resistance against mildew. Unlike mycotoxins, the content of ergosterol increases (*P* < 0.05) from October to December. It is obvious that the higher humidity of the growing season contributes to the development of mould, but low temperatures inhibit formation of mycotoxins. Fall of temperature under 5 °C in November and December can lead to reduction of enzymatic activity of mould and lower production of secondary metabolites, particularly mycotoxins, which comprise stress reaction on the higher temperature. On the contrary temperatures lower than 5 °C are favourable for the growth of some moulds (*Fusarium nivale*) and this can enhance the content of ergosterol without increase of mycotoxins.

## 4. Conclusions

During the growing season forage grasses can become contaminated with mycotoxins, most of all by deoxynivalenol (DON) and zearalenone (ZEA). This phenomenon mainly occurred in July and in October, which means a high risk of mycotoxin input to the food chain. Differences in safety of individual grass species were not statistically proven, although the lower content of ZEA in *Festulolium pabulare* was evident.

## Figures and Tables

**Table 1:** Changes in the content of ergosterol (ERG), zearalenone (ZEA) and deoxynivalenol (DON).

Factor	Deoxynivalenol		Zearalenone		Ergosterol	

	x (ppb)	s.e.	x (ppb)	s.e.	x (mg kg^−1^)	s.e.

**Specie**						

*Lolium perenne*	46.02	10.83	34.06	30.87	76.61 ^ab^	29.80
*Festulolium pabulare*	37.78	9.61	9.82	5.05	35.65 ^a^	13.12
*Festulolium braunii*	33.83	9.06	72.85	48.53	82.53 ^b^	32.55
*Mixture with Festuca rubra*	46.60	11.54	91.81	61.18	71.71 ^ab^	24.30
*Mixture with Poa pratensis*	45.55	13.25	96.24	59.50	68.05 ^ab^	23.74

**Date of cut**						

June	12.34 ^a^	5.07	0.0 ^a^	0.01	3.84 ^a^	0.92
July	71.43 ^b^	7.67	122.3 ^ab^	62.54	13.07 ^a^	3.72
October	52.95 ^bc^	9.16	173.0 ^b^	66.17	31.87 ^a^	3.91
November	51.71 ^bc^	12.04	3.7 ^a^	3.59	94.94 ^b^	11.31
December	21.35 ^ac^	6.07	5.8 ^a^	3.82	190.82 ^c^	23.89

**Year**						

2008	37.63	7.66	115.76 ^a^	37.82	68.50	19.18
2009	46.28	5.67	6.15^b^	2.50	65.31	12.05

s.e. standard error; x mean; mean values in the same column with different superscripts (a, b, c) are significant at a level of *P* < 0.05.

## References

[1] Reverberi M Ricelli A Zjalic S Fabbri AA Fanelli C Natural functions of mycotoxins and control of their biosynthesis in fungi Appl. Microbiol. Biotechnol 2010 87 899 911 20495914 10.1007/s00253-010-2657-5

[2] Behrendt U Muller T Seyfarth W The influence of extensification in grassland management on the populations of micro-organisms in the phyllosphere of grasses Microbiol. Res 1997 152 75 85

[3] Holubek R Holubek I Stehlikova B The influence of various ways of regeneration and fertilization on economic profit from grass stand Acta Fytotechnica et Zootechnica 2001 5 74 77

[4] Giesler LJ Yuen GY Horst GL The microclimate in tall fescue turf as affected by canopy density and its influence on brown patch disease Plant Dis 1996 80 389 394

[5] Skladanka J Adam V Ryant P Dolezal P Havlicek Z Can be *Festulolium*, *Dactylis glomerata* and *Arrhenatherum elatius* used for extending autumn grazing season in Central Europe? Plant Soil Environ 2010 56 488 498

[6] von Boberfeld WO Banzhaf K Hrabe F Skladanka J Kozlowski S Golinski P Szeman L Tasi J Effect of different agronomical measures on yield and quality of autumn saved herbage during winter grazing—2(nd) communication: Crude protein, energy and ergosterol concentration Czech J. Anim. Sci 2006 51 271 277

[7] von Boberfeld WO Changes of the quality including mycotoxin problems of the primary growth of a hay meadow—Arrhenatherion elatioris. Agribiol. Res.-Z. Agrarbiol. Agrik.chem. Okol 1996 49 52 62

[8] Leitao AL Potential of penicillium species in the bioremediation field Int. J. Environ. Res. Public Health 2009 6 1393 1417 19440525 10.3390/ijerph6041393 PMC2681198

[9] Sammon NB Harrower KM Fabbro LD Reed RH Microfungi in drinking water: The role of the frog Litoria caerulea. Int. J. Environ. Res. Public Health 2010 7 3225 3234 10.3390/ijerph7083225 PMC2954578 20948957

[10] von Boberfeld WO Banzhaf K Yield and forage quality of different x*Festulolium* cultivars in winter J. Agron. Crop Sci 2006 192 239 247

[11] Skladanka J Dohnal V Dolezal P Jezkova A Zeman L Factors affecting the content of ergosterol and zearalenone in selected grass species at the end of the growing season Acta Vet. BRNO 2009 78 353 360

[12] Marin S Vinaixa M Brezmes J Llobet E Vilanova X Correig X Ramos AJ Sanchis V Use of a MS-electronic nose for prediction of early fungal spoilage of bakery products Int. J. Food Microbiol 2007 114 10 16 17207549 10.1016/j.ijfoodmicro.2006.11.003

[13] Vasatkova A Krizova S Adam V Zeman L Kizek R Changes in metallothionein level in rat hepatic tissue after administration of natural mouldy wheat Int. J. Mol. Sci 2009 10 1138 1160 19399242 10.3390/ijms10031138 PMC2672023

[14] Vasatkova A Krizova S Krystofova O Adam V Zeman L Beklova M Kizek R Effect of naturally mouldy wheat or fungi administration on metallothioneins-3 level in brain tissues of rats Neuroendocrinol. Lett 2010 30 163 168 20027165

[15] Dohnal V Jezkova A Jun D Kuca K Metabolic pathways of T-2 toxin Curr. Drug Metab 2008 9 77 82 18220574 10.2174/138920008783331176

[16] Wu QH Dohnal V Huang LL Kuca K Yuan ZH Metabolic pathways of trichothecenes Drug Metab. Rev 2010 42 250 267 19678805 10.1080/03602530903125807

[17] Kushiro M Effects of milling and cooking processes on the deoxynivalenol content in wheat Int. J. Mol. Sci 2008 9 2127 2145 19330063 10.3390/ijms9112127 PMC2635633

[18] Ueno Y The toxicology of mycotoxins CRC Crit. Rev. Toxicol 1985 14 99 132 10.3109/10408448509089851 3158480

[19] Ueno Y Toxicology of trichothecene mycotoxins ISI Atlas Sci.-Pharm 1988 2 121 124

[20] Sobrova P Adam V Vasatkova A Beklova M Zeman L Kizek R Deoxynivalenol and its toxicity Interdisc. Toxicol 2010 3 101 106 10.2478/v10102-010-0019-x PMC2984136 21217881

[21] Kumar V Basu MS Rajendran TP Mycotoxin research and mycoflora in some commercially important agricultural commodities Crop Prot 2008 27 891 905

[22] Mngadi PT Govinden R Odhav B Co-occurring mycotoxins in animal feeds Afr. J. Biotechnol 2008 7 2239 2243

[23] Niessen L PCR-based diagnosis and quantification of mycotoxin producing fungi Int. J. Food Microbiol 2007 119 38 46 17804102 10.1016/j.ijfoodmicro.2007.07.023

[24] Sarmadha MK Balachandran C Serum electrolyte changes in Penicillic acid toxicosis Indian Vet. J 2008 85 248 250

[25] Wagacha JM Muthomi JW Mycotoxin problem in Africa: Current status, implications to food safety and health and possible management strategies Int. J. Food Microbiol 2008 124 1 12 18258326 10.1016/j.ijfoodmicro.2008.01.008

[26] Annison EF Bryden WL Perspectives on ruminant nutrition and metabolism I. Metabolism in the rumen Nutr. Res. Rev 1998 11 173 198 19094246 10.1079/NRR19980014

[27] Schatzmayr G Zehner F Taubel M Schatzmayr D Klimitsch A Loibner AP Binder EM Microbiologicals for deactivating mycotoxins Mol. Nutr. Food Res 2006 50 543 551 16715543 10.1002/mnfr.200500181

[28] Gutzwiller A Czegledi L Stoll P Bruckner L Effects of *Fusarium* toxins on growth, humoral immune response and internal organs in weaner pigs, and the efficacy of apple pomace as an antidote J. Anim. Physiol. Anim. Nutr 2007 91 432 438 10.1111/j.1439-0396.2006.00672.x 17845251

[29] Leung MCK Smith TK Karrow NA Boermans HJ Effects of feedborne *Fusarium* mycotoxins with and without a polymeric glucomannan mycotoxin adsorbent on body weight, feed intake, serum chemistry, and nutrient digestibility of mature beagles Poult. Sci 2007 86 267 26817234839

[30] Kurtbay HM Bekci Z Merdivan M Yurdakoc K Reduction of ochratoxin A levels in red wine by bentonite, modified bentonites, and chitosan J. Agric. Food Chem 2008 56 2541 2545 18321048 10.1021/jf073419i

[31] Worrell NR Mallett AK Cook WM Baldwin NCP Shepherd MJ The Role of Gut Microorganisms in the Metabolism of Deoxynivalenol Administered to Rats Xenobiotica 1989 19 25 32 2756716 10.3109/00498258909034673

[32] Baird R Abbas HK Windham G Williams P Baird S Ma P Kelley R Hawkins L Scruggs M Identification of select fumonisin forming *Fusarium* species using PCR applications of the polyketide synthase gene and its relationship to fumonisin production in vitro Int. J. Mol. Sci 2008 9 554 570 19325769 10.3390/ijms9040554 PMC2635686

[33] Guan S Ji C Zhou T Li JX Ma QG Niu TG Aflatoxin B-1 degradation by *Stenotrophomonas maltophilia* and other microbes selected using coumarin medium Int. J. Mol. Sci 2008 9 1489 1503 19325817 10.3390/ijms9081489 PMC2635738

[34] Mantle PG Nagy JM Binding of ochratoxin a to a urinary globulin: A new concept to account for gender difference in rat nephrocarcinogenic responses Int. J. Mol. Sci 2008 9 719 735 19325780 10.3390/ijms9050719 PMC2635713

[35] Martins HM Almeida I Marques M Bernardo F Interaction of wild strains of Aspergilla with *Aspergillus parasiticus* ATCC15517 on aflatoxins production Int. J. Mol. Sci 2008 9 394 400 19325757 10.3390/ijms9030394 PMC2635674

[36] Lancova K Hajslova J Kostelanska M Kohoutkova J Nedelnik J Moravcova H Vanova M Fate of trichothecene mycotoxins during the processing: Milling and baking Food Addit. Contam 2008 25 650 659 10.1080/02652030701660536 18473219

[37] Dohnal V Jezkova A Pavlikova L Musilek K Jun D Kuca K Fluctuation in the ergosterol and deoxynivalenol content in barley and malt during malting process Anal. Bioanal. Chem 2010 397 109 114 20225055 10.1007/s00216-010-3585-z

[38] Skladanka J Dohnal V Jezkova A Fibre and ergosterol contents in forage of *Arrhenatherum elatius*, *Dactylis glomerata* and *Festulolium* at the end of the growing season Czech J. Anim. Sci 2008 53 320 329

[39] de Nijs M Soentoro P Asch EDV Kamphuis H Rombouts FM Notermans SHW Fungal infection and presence of deoxynivalenol and zearalenone in cereals grown in the Netherlands J. Food Prot 1996 59 772 777 10.4315/0362-028X-59.7.772 31159090

[40] Engels R Kramer J Incidence of fusaria and occurence of selected *Fusarium* mycotoxins on *Lolium* ssp. in Germany Mycotoxin. Res 1996 12 31 40 10.1007/BF03192078 23604632

[41] Dmello JPF Macdonald AMC Mycotoxins Anim. Feed Sci. Technol 1997 69 155 166

[42] Marasas WFO Vanrensburg SJ Mirocha CJ Incidence of *Fusarium* species and the mycotoxins, deoxynivalenol and zearalenone, in corn produced in esophageal cancer areas in transkei J. Agric. Food Chem 1979 27 1108 1112 161914 10.1021/jf60225a013

